# Mortality for Critical Congenital Heart Diseases and Associated Risk Factors in Newborns. A Cohort Study

**DOI:** 10.5935/abc.20180175

**Published:** 2018-11

**Authors:** Selma Alves Valente do Amaral Lopes, Isabel Cristina Britto Guimarães, Sofia Fontes de Oliva Costa, Angelina Xavier Acosta, Kyoko Abe Sandes, Carlos Maurício Cardeal Mendes

**Affiliations:** 1 Departamento de Pediatria, Faculdade de Medicina da Bahia, Universidade Federal da Bahia (UFBA), Salvador, BA - Brasil; 2 Programa de Pós Graduação em Processos Interativos dos Órgãos e Sistemas, Instituto de Ciências da Saúde, Universidade Federal da Bahia (UFBA), Salvador, BA - Brasil; 3 Instituto de Ciências da Saúde, Universidade Federal da Bahia (UFBA), Salvador, BA - Brasil; 4 Faculdade de Medicina da Bahia, Universidade Federal da Bahia (UFBA), Salvador, BA - Brasil

**Keywords:** Heart Defects Congenital/mortality, Infant Newborn/mortality, Risk Factors, Survival Analysis

## Abstract

**Background:**

Congenital heart diseases are the most common type of congenital defects, and account for more deaths in the first year of life than any other condition, when infectious etiologies are ruled out.

**Objectives:**

To evaluate survival, and to identify risk factors in deaths in newborns with critical and/or complex congenital heart disease in the neonatal period.

**Methods:**

A cohort study, nested to a randomized case-control, was performed, considering the Confidence Interval of 95% (95% CI) and significance level of 5%, paired by gender of the newborn and maternal age. Case-finding, interviews, medical record analysis, clinical evaluation of pulse oximetry (heart test) and Doppler echocardiogram were performed, as well as survival analysis, and identification of death-related risk factors.

**Results:**

The risk factors found were newborns younger than 37 weeks (Relative Risk - RR: 2.89; 95% CI [1.49-5.56]; p = 0.0015), weight of less than 2,500 grams (RR: 2.33 [; 95% CI 1.26-4.29]; p = 0.0068), occurrence of twinning (RR: 11.96 [95% CI 1.43-99.85]; p = 0.022) and presence of comorbidity (RR: 2.27 [95% CI 1.58-3.26]; p < 0.0001). The incidence rate of mortality from congenital heart disease was 81 cases per 100,000 live births. The lethality attributed to critical congenital heart diseases was 64.7%, with proportional mortality of 12.0%. The survival rate at 28 days of life decreased by almost 70% in newborns with congenital heart disease. The main cause of death was cardiogenic shock.

**Conclusion:**

Preterm infants with low birth weight and comorbidities presented a higher risk of mortality related to congenital heart diseases. This cohort was extinguished very quickly, signaling the need for greater investment in assistance technology in populations with this profile.

## Introduction

Before the age of cardiac surgery, less than 50 years ago, just over 30% of children with severe Congenital Heart Diseases (CHD) survived into adulthood. This change was due to the evolution not only in the technique of cardiac surgery, and adaptation of cardiac catheterization to newborns, but also in the anesthetic technique, as well as the improvements in neonatal and pediatric intensive care units. Thus, the countries that have organized their care network, following this evolution pattern, have been able to considerably increase survival with quality of life for children with severe CHD. In these countries, mortality from heart disease has dropped dramatically, with up to 85% of these newborns surviving adulthood.^1^^-^^3^

In spite of all this progress, CHDs are related to increased fetal losses,^4^ being present in up to 85% of the deaths in necropsy findings in stillbirths, newborns, and infants,^5^ being the main cause of cardiac arrest up to 24 years of age, ranging from 84% in the first two years to 21% in the second decade of life.^6^

In addition, CHD mortality has a great variability worldwide. Low-industrialized or developing countries, where access to health is precarious, have substantially higher mortality rates than developed countries, which are consistent with national studies.^7^^-^^9^

In the statistics with more methodological strictness, it is expected that, for serious heart diseases, such as conotruncal defects, tetralogy of Fallot, transposition of large arteries, and truncus arteriosus, survival in the first year of life fluctuates from 62.8% to 79, 6%, with a worse result for truncus arteriosus.^10^ For hypoplastic left heart syndrome, data are more discouraging even in the main centers, with neonatal mortality of 68%, and mortality up to 3 months of 81%, depending on the moment that this newborn is seen. The later the care in a reference center, the greater the mortality.^6^^,^^11^

The literature indicates that premature newborn infants with a low Apgar score, and who require invasive ventilatory support, are those who present higher risk of mortality when more complex procedures are required.^12^^,^^13^

The objective of this article was to describe the mortality, fatality, and survival rates of CHD newborns in a Brazilian large urban center, as well as to characterize the associated risk and morbidity factors.

## Methods

A nested case-control study cohort was performed, paired with newborns selected by lot, born in the city of Salvador (state of Bahia) and in its respective metropolitan region, from December 2014 to January 2016. The original sample was a case-control study paired by maternal age and newborn age, in which 52 cases of critical and complex CHD were selected in the neonatal period.

Data were prospectively collected in the four largest public maternity hospitals in the city of Salvador. All newborns were placed in the process of regulation to a specialized center, but they did not undergo any interventional procedure until transfer, since none of the maternities had a cardiac surgery service. The follow-up and the recording of newborns monitoring were performed up to the moment of discharge from the maternity ward (due to clinical improvement, transference or death).

The independent variables were: gestational age, low birth weight (weight less than < 2,500 grams), pulse oximetry test (POT), cardiac auscultation (presence or absence of murmur or irregular heart rhythm), Apgar, twinning, and presence of comorbidities (neonatal sepsis, and respiratory insufficiency, with demand for invasive ventilatory support).

The dependent variable was the occurrence of critical and/or complex CHD, and the secondary outcome was death.

The CHD cases included were the critical CHD newborns, which were channel-type or shunt-dependent, or considered complex (those with three or more defects), born in the services included in the study, in the reported period. For the comparison group, the neonates without CHD were included, selected by lot, of the same gender of the case, with more than 24 hours of life who, on physical examination, did not present murmurs or arrhythmias, with pre- and post-ductal oximetry, and differential not exceeding 3% and above 95% saturation.

Considering a possible fallibility of POT, and aiming at minimizing possible losses, these newborns were followed by telephone or at the childcare outpatient clinic up to 3 months after discharge from the maternity ward. In addition, in order to minimize possible losses, and to identify allocation errors, in the first year after completion of data collection, all newborns and infants entries were monitored in the only public high-complexity pediatric cardiac surgery service of the state of Bahia.

Newborns whose only identified heart disease was the presence of Patent Arterial Duct (PAD), or other simple heart diseases; with pulmonary hypertension without structural heart disease; cases that were not characterized as CHD; newborns whose parents or guardians did not sign the Free and Informed Consent Form (FICF) were excluded from the study.

This study was approved by the Research Ethics Committee (CEP) of Hospital Ana Nery and by the local Ethics Committees of each hospital involved (CAAE: 17970413200000045). The FICF was used to make the child’s legal guardian aware of the process.

For the proportional mortality calculations, mortality data were used in the neonatal period, for the same sampled population and period studied.

Sample size estimation was performed primarily for the case-control study, considering the proportion of exposed cases within of 20%; proportion of exposed, among controls/comparison group of 11.11%,%; Odds Ratio (OR) 2; and significance level of 5% (test power: 80%).

### Statistical analysis

For the direct estimation of gross relative risks, we chose to perform simple Poisson regression modeling, associated to the robust estimation of standard errors, aiming to control some possible average violation of the assumption of equality between mean and variance of the distribution of Poisson, and consequent more adequate estimation of the model p values, and level of significance of 5%.^14^ For the calculation of the Confidence Intervals of 95% (95% CI), the use of the Delta 2 method was added. The model goodness of fit was evaluated by analyzing the residual *deviance* and the *Akaike* Information Criterion (AIC).^15^

In the Kaplan-Meier survival curves analysis, Cox regression modeling with right censorship was used to obtain survival probability and hazard ratio (HR), assuming proportionality risk. For the comparison of the survival curves, Log rank test was used. The database was created in Epidata,^16^ version 3.1, and the statistical analyzes were performed in the statistical package R, version 3.2.3.^17^

## Results

Fifty-two cases of CHD newborns with critical and complex congenital heart disease and their respective comparison groups, in the maternity hospitals studied, were identified and monitored. The most frequent heart diseases were formation of aortic arch defects, which depended on the ductus arteriosus (62 cases/100,000 live births), followed by pulmonary atresia with or without hypoplasia of the right ventricle (53 cases/100,000 births), and transposition of the great arteries (38 cases/100,000 live births).

As a consequence of gender pairing, the distribution was equal between the groups (OR: 0.92; 95% CI: 0.67-1.27]). In the initial data, there was one case of ambiguous genitalia; however, during follow-up it was confirmed that it was a female newborn.

The risk of death among newborn infants with CHD was twice as high among premature infants (RR: 2.14; 95% CI [1.22-3.75]; p = 0.003), with low birth weight (RR: 2.14; 95% CI [1.22-3.75]; p < 0.0001) and Apgar < 7 in the first minute of life (RR: 2.08; 95% CI [1.13-3.82]; p = 0.017). The presence of some comorbidity, besides CHD, was associated with the outcome, and increased the risk by almost three times (p < 0.0001). There was a higher proportion of twins among the cases (9.9%) (RR: 13.1; 95% IC [1.59-109.1]; p = 0.018) than newborns without heart disease (2.2%), and for this condition, the risk of death was 12 times higher among twin newborns with CHD (Table 1).

**Table 1:** Association between congenital heart disease and factors related to the newborn

Variable	Factor	RR *	CI 95%	p value	AIC§
Gender	Female	1		-	
	Male	0.92	0.66-1.27	0.6	301.2
Weight	Above > 2,500 g	1		-	
	Low Weight (< 2,500 g)	2.33	1.26-4.29	0.0068	170.5
Gestational Age	> 37 weeks	1		-	
	< 37 weeks	2.89	1.49-5.56	0.0015	157.9
Apgar 1st minute	≥ 7	1		-	
	Less than < 7	2.35	1.25-4.45	0.0084	163.1
Apgar 5^th^ minute	≥ 7	1		-	
	<7	9.49	1.09-82.85	0.042	43.9
Twinning	No	1		-	
	Yes	11.96	1.43-99.85	0.022	48.8
Heart auscultation alteration	Normal	1		-	
	Changed	84	11.83-596.21	< 0.0001	112.6
Comorbidities	No	1		-	
	Yes	2.27	1.58-3.26	< 0.0001	215.5

* Gross RR by Poisson regression; p value - Z statistic. RR: relative risks; 95% CI: 95% confidence interval; AIC: Akaike Information Criterion.

**Figure 1:**
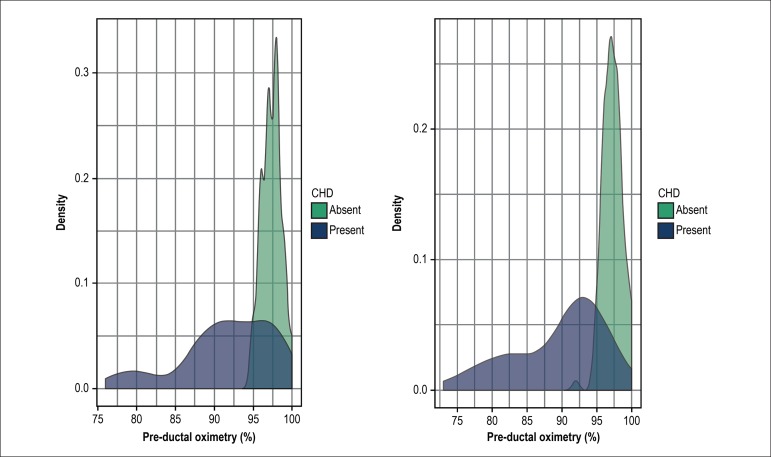
Distribution of recording density of pre- and post-ductal pulse oximetry levels, according to the presence or absence of congenital heart diseases (CHD).

Clinical data on changed cardiac auscultation were found in 72% of cases and in only 1% of infants without CHD. The difference of this finding was related to the higher risk for CHD (p < 0.0001). Pulse oximetry was recorded even for cases of CHD with intrauterine diagnosis or in those in which another finding was the clinical suspicion and where the diagnosis had been made before 24 hours of life. Figure 1illustrates the differential distribution density of pulse oximetry measurements among newborns with and without CHD. Some records below the cut-off level are noted for newborns without CHD, for whom the echocardiogram was required, and the possibility of CHD was ruled out.

The incidence of death in CHD cases was 81/100 thousand live births. The case fatality rate attributed to CHD was 64.7%, with proportional mortality of 12.0% (17/142). The main cause of death was cardiogenic shock in 41.1% of the cases, followed by sepsis (17.6%) in three newborns with Double Right Ventricular Outflow Tract (DRVOT), and impossibility of therapy for cardiopathy (17.6%) - CHD anatomy was not consistent with any surgical procedure available, progressing to refractory hypoxemia followed by death - in neonates with hypoplastic left heart syndrome and untreatable ill-defined cardiac defects (Table 2).

**Table 2:** Causes of death, according to the type of cardiopathy

Type of cardiopathy	Cause of death	n (%)
PVA, IVCa, PVAD, HLV, GAT, AVI, and TrA	Cardiogenic shock	7 (41,1)
Ebstein's anomaly	Supraventricular tachycardia	1 (5,9)
RVDO	Sepsis	3 (17,6)
LHHS, pentalogy of Cantrell	Through CHD (basic cause/palliative care)	3 (17,6)
PVAD, GAT	Ill-defined causes	3 (17,6)

PVA: post-varicella angiopathy; IVC: interventricular communication; PVAD: Pulmonary vein anomalous drainage; HLV: hypoplasic left ventricle; GAT: great arteries transposition; AVI: aortic valve insufficiency; TrA: Truncus arteriosus; RVDO: right ventricle double outlet; CHD: congenital heart disease.

**Figure 2:**
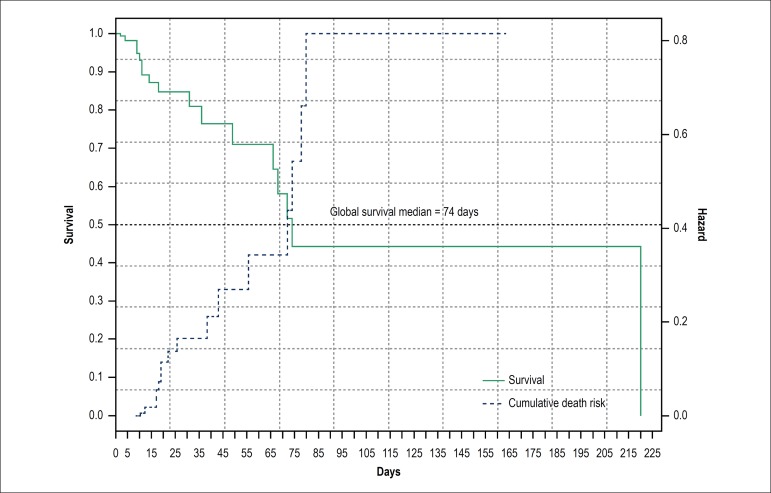
Kaplan-Meier Curves and Cumulative Risk Function for Global Mortality for Congenital Heart Diseases.

The median hospital stay was 75 days, with an increased risk of death of 0.4 to 0.8 (HR: 0,4->0.8). Still in the neonatal period, 25% of CHD newborns had already died (Figure 2).

**Figure 3:**
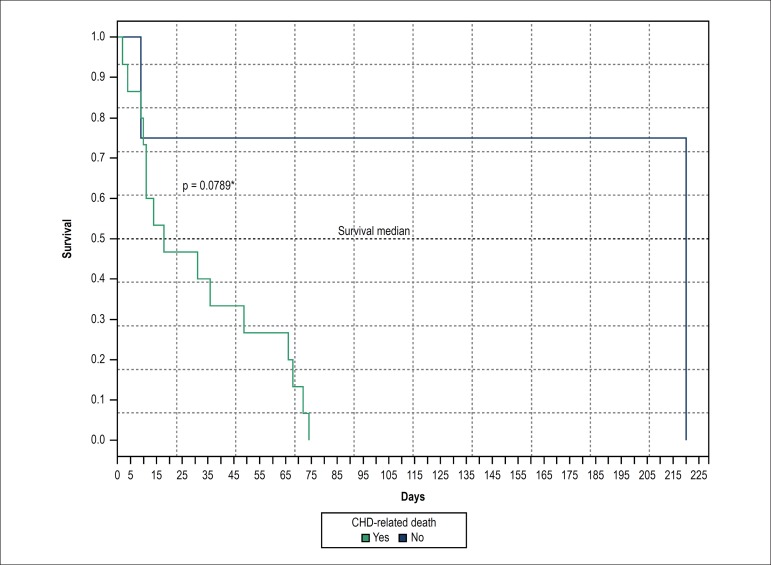
Kaplan-Meier curves, according to deaths related or not to heart disease. CHD: congenital heart defects.

There was no statistical difference for survival rates when the death event was compared between those who died from CHD and due to other causes (p = 0.076). Although survival in these newborns has declined by more than 50% in the first 10 days of life and within the neonatal period, this survival declined by more than 60% (Figure 3) before newborns achieved 28 days of life.

## Discussion

CHD newborns presented higher morbidity attributed to prematurity, low birth weight, some degree of intrauterine fetal distress, both due to physical examination and changed pulse oximetry. The literature has drawn attention to the greater morbidity, especially of premature newborn infants, who already present a range of other pathologies due to their constitution, which can substantially aggravate these patients progress.^12^

For both post-ductal (RR: 46; 95% CI [11.54-184.0]) and pre-ductal (RR: 39; 95% CI [9.72-157.5]) variable oximetry, the differences between groups were well established. This data not only reinforced the validation of the controls, but also confirmed the importance of making this screening test universal. On the other hand, the physical examination had low specificity (40%) and regular sensitivity, a little higher than the POT (89%), but, alone, it was insufficient to rule out the possibility of CHD. The literature states that when the physical examination is performed by a well-trained and experienced pediatrician, there is an increase in the sensitivity of the POT by up to 20%,^18^ optimizing the detection capacity when they are appropriately associated.^19^^,^^20^

The finding of a low Apgar score in the first minute denoted the importance of knowing that some cardiopathies may be active in the uterus, impairing the blood flow that would allow adequate supply of nutrients and oxygen to the fetus, which may affect the morbidity and mortality of this newborn; this reinforces the importance of adequate prenatal diagnosis and follow-up. Studies in Brazil have already indicated that low access to prenatal and/or at birth diagnosis makes the treatment of CHD considerably difficult, which leads to a worse clinical condition at birth.^9^

The frequency of twin pregnancies among the cases was proportionally higher within the comparison group. This data was reported with controversy in other studies, due to the difficulty of concomitantly evaluating the association of other risk factors, but for the outcome death, this finding was determinant.^21^

The early and high mortality rate found here was one of the most discordant data in the world literature. In developed countries, it is expected that the CHD fatality in the neonatal period will only exceed 60% for the late diagnoses of the hypoplastic left heart syndrome (HLHS); for the other types of CHD, the expected fatality rate does not exceed 40%, when the diagnosis of CHD is made before hospital discharge.^22^ Countries with socioeconomic classification similar to that of Brazil, although also coping with glaring regional differences in relation to neonatal care, have an overall incidence rate of CHD deaths of 20 to 30/100,000 births.^2^ Fixler et al.^3^ measured the mortality rate according to the time of referral, considering first day, up to 5 days, 4 to 27 days, and no referral after 27 days, and found mortality near 38% when the newborn was not referred before 27 days of life. In addition, mortality increased considerably at 3 months, getting close to 80% for HLHS.^3^

The literature has shown a significant improvement in the quality of care, which has led to a decrease in morbidity and mortality in developed countries,^3^^,^^4^ but this is not a reality for developing countries, as can be seen in the high mortality and lethality rate despite the same incidence of CHD described herein.

Neonatal deaths due to congenital defects are classified by some authors,^23^ and by the Brazilian Ministry of Health as avoidable, because they may be reduced for some conditions, if adequate and prompt assistance is offered to the pregnant woman and the newborn, aiming at the diagnosis and treatment, associated with adequate support by other spheres of the government - other than health services.^24^ In addition, pathologies with this classification have the possibility of reducing mortality by such actions, depending on the condition considered.^25^^,^^26^

The Ministry of Health recently launched a project to extend care to CHD children,^27^ to reduce the mortality from these defects, which is in agreement with the findings in this study. This mobilization was necessary because it was estimated, within the national context, that up to 80% of newborns with CHD require a surgical procedure at some point in their development. Not infrequently, there is some demand for a surgical approach until late adolescence and early adulthood.^28^ These data, while they may be underestimated,^29^ should be monitored by independent and prospectively validated scientific investigations as the policy in question is being implemented.

### Limitations of the study

Although the minimum sample size was calculated, considering the local prevalence of CHD in a pilot study, some variables could not be included in the regression model due to the numerical insufficiency, a consequence of the multivariate approach. The absence of statistical difference for survival rates, when the death event was compared within those who died from CHD and other causes (p = 0.076), is possibly related to the numerical insufficiency of this subgroup. In addition, in the period from September 2015 to January 2016, there was a substantial reduction in the number of occurrences of CHD from not yet well specified causes (data from the Department of Information Technology of the National Unified Health System - DATASUS, and direct observation in the collection of data), which resulted in longer collection time.

## Conclusion

The high lethality rate of the disease in question demands critical attention for structuring a specialized care network, which can adequately serve the volume of neonates with congenital heart disease, as well as provide real investments in training and care technology, even within the neonatal age group. As an example, we can cite the policies that are directed to actions, aiming to deepen the scientific knowledge about the cardiopathies and their clinical interpellations.

The neonatal mortality rate from critical congenital heart diseases was higher in this study than in countries with the same economic classification. In addition, this cohort was very quickly extinguished, which is very concerning, considering that death was the main outcome in very young patients, who did not have the opportunity to receive the specialized treatment. These findings point to the need for greater investment in care technology in populations with this profile.
